# Black and Minoritized Women’s Experiences of Specialist Domestic Violence Services in the United Kingdom (UK): A Scoping Review

**DOI:** 10.1177/15248380251335038

**Published:** 2025-04-24

**Authors:** Penelope E. Lowe, Sally McManus, Pardis Asadi Zeidabadi, Ravi K. Thiara, Sumanta Roy, Estela Capelas Barbosa, Ladan Hashemi

**Affiliations:** 1 University of Roehampton, London, UK; 2 City St George’s, University of London, London, UK; 3 National Centre for Social Research, London, UK; 4 University of Warwick, Coventry, UK; 5 Imkaan, London, UK; 6 University of Bristol, Bristol, UK

**Keywords:** Black and minoritized women, domestic violence and abuse, service provision, intersectionally competent, specialist, “by and for” services

## Abstract

This scoping review maps the existing available literature on Black and minoritized women’s experiences with specialist Domestic Violence and Abuse (DVA) services in the UK to summarize current understanding and identify knowledge gaps. A comprehensive search was conducted across multiple databases and gray literature sources. All articles had to include Black and minoritized women’s experiences of DVA services. In total, 29 UK-based studies published between 2000 and 2024 were identified. Data were synthesized to identify key themes and gaps. Thematic analysis of the findings revealed three main themes: additional service needs, barriers to accessing support, and the pivotal role of “by and for” services. Our review concludes that “by and for” services—provided by and for minoritized women—which adopt an intersectional approach are crucial in addressing the unique needs of Black and minoritized “survivor–victims”, particularly in terms of language support, practical assistance, and community-related support. There is a need for more peer-reviewed literature to recognize the role of “by and for” services, using diverse methodologies to support Black and minoritized communities better.

## Introduction

Domestic violence and abuse (DVA) harms individuals from all backgrounds, yet the experiences and needs of different communities vary significantly. The Crime Survey for England and Wales (CSEW) estimated that over 1.4 million women and 751,000 men experienced DVA in the year ending March 2023. This survey revealed higher proportions of self-reported DVA among White women (6.0%) compared to Black (3.1%) or Asian (3.0%) women (Office for National Statistics, 2023). Although these statistics suggest a lower prevalence of reported DVA among Black and minoritized groups, it is vital to acknowledge the unique barriers to reporting and seeking help faced by these communities that may also affect participation and reporting in surveys (Femi-Ajao et al., 2018).

Black and minoritized women may face heightened vulnerability to DVA due to factors such as socio-economic deprivation, racism, and exposure to specific forms of violence such as so-called honor-based abuse (HBA), female genital mutilation (FGM) or forced marriage (Gill & Walker, 2020; Pittman et al., 2020; Slabbert, 2016). In addition, minoritized “survivor–victims” of DVA may face intersecting challenges within their socio-political and community context that impede their ability to disclose DVA (Asante, 2024). For example, they may experience social stigma, community pressures, unmet need for social support, isolation, hostile immigration policies, and fears of racism from service providers (Asante, 2024; Hulley et al., 2022).

In response to these challenges, there is increasing recognition of the need for intersectionally informed interventions. It is pivotal that interventions for Black and minoritized DVA “survivor–victims” move beyond assumptions of culture to the broader intersectional context of victimization and vulnerability. Cultural framing leads to the stereotyping and stigmatization of certain communities while neglecting the broader structural inequalities that perpetuate DVA practices (Justice Analytical Services [JAS], 2024).

DVA services rooted in a Eurocentric and non-intersectional model may inadequately address the diverse needs of minoritized DVA “survivor–victims” (Asante, 2024). In contrast, intersectionally informed services are tailored to meet the diverse and unique needs of minoritized women, including community-related needs, language and financial support, and health-related needs. Evidence from international contexts suggests that intersectionally tailored programs are highly effective. For example, studies in the United States (US) have demonstrated the success of tailored programs for Latina “survivor–victims”.

Analyses of three different Latino specialist DVA service providers found appropriate responses to unique Latina (women’s) needs (Fuchsel, 2014; Fuchsel & Hysjulien, 2013; Perilla et al., 2012; Rizo & Macy, 2011), including significant trauma-related symptoms, language barriers, fear of deportation, and an inability to recognize abusive behaviors within a patriarchal community (Edelson et al., 2007; Reina et al., 2013). A flexible, holistic approach responded to Latinas’ reluctance to engage in individual therapy by developing a non-hierarchical group format to promote equal and respectful relationships (Perilla et al., 2012). The empowerment and effectiveness of support provided were correlated with the level of contextual tailoring to meet the specific needs of minoritized service users (Fuchsel, 2014; Fuchsel & Hysjulien, 2013; Perilla et al., 2012). Similar successes have been observed with First Nation women in Canada (Klingspohn, 2018) and Aboriginal women in Australia (Pokharel et al., 2021).

The topic of DVA services specialized for minoritized women is under-researched in the UK compared to the growing body of peer-reviewed international literature. The existing studies have investigated the unique needs of Black and minoritized DVA “survivor–victims” within the UK. For example, a scoping review by Femi-Ajao et al. (2018) emphasized the importance of integrating faith-based resources into DVA services for Black and minoritized communities. The barriers to help-seeking for minoritized communities, particularly the significant legal and policy barriers for immigrant women (Anitha, 2011; Burman & Chantler, 2005), have also been documented. Despite the established need for intersectionally informed services in the UK, the academic literature has yet to thoroughly explore the lived experiences of Black and minoritized women.

The DVA support sector in the UK employs a multi-agency approach to provide refuges, housing, mental health and therapeutic services, migration support, legal support, and helplines across multiple individual agencies. The “by and for” service sector—run by and for minoritized women—is central to service provision for Black and minoritized DVA “survivor–victims”, as the support is specifically designed to meet their intersecting needs (Imkaan, 2015; The Equality and Human Rights Commission, 2020). The term “intersectionally informed service” applies to “by and for” services, reflecting the tailored support provided to meet the practical, language, and community-related needs of minoritized women. However, “by and for” services are often local, grassroots services with poor recognition from the UK government or commissioning bodies (Imkaan, 2015).

### The Present Study

Given the distinctive characteristics of the UK’s DVA service sector, this review explores the evidence on Black and minoritized women’s experiences of specialist DVA services in the UK. Specifically, it examines Black and minoritized women’s experiences with specialist “by and for” services and broader DVA services. This scoping review seeks to map existing evidence and research, identify gaps in current understanding, and assess how minoritized women in the UK experience specialist DVA services.

## Methods

The review was conducted following Arksey and O’Malley’s (2005) methodological framework for scoping reviews, which details identifying the research question, locating relevant studies, selecting studies for inclusion, charting the data, and finally collating, summarizing, and reporting the results. This framework ensures a comprehensive exploration of the topic, especially useful when high variability in terminology and methods exists. In writing the report, the Preferred Reporting Items for Systematic Reviews and Meta-Analyses Extension for Scoping Reviews (PRISMA-ScR) Checklist (Tricco et al., 2018) was followed.

### Definitions

This review used the Domestic Abuse Act 2021 definition of domestic abuse: abusive behavior (i.e., psychological, economic, coercive behavior, physical, sexual or violent behavior, and abuse, regardless of whether it is a single incident or repeated) from one person to another, who are personally connected (i.e., victim-perpetrator were/are married or civil partners, engaged, relatives, partners, have a child together). This definition also encompasses forced marriage, HBA, and instances of FGM when committed or arranged by a personally connected individual.

This review uses the term “survivor–victims” of DVA to refer to women who have experienced any form of DVA at any point. While anyone can experience DVA, this review focuses on female “survivor–victims” due to the prevalence of DVA experience amongst this group (Imkaan & Centre for Women’s Justice, 2023). Black and minoritized is used to refer to ethnic groups that have been actively minoritized through social processes and White centering (Selvarajah et al., 2020). Due to the limitations of the included literature, which often could not distinguish between different ethnic groups because of sample size constraints, this review was also unable to draw any distinction between specific ethnic groups. Therefore, we use the term “Black and minoritized” to refer to participants from various marginalized backgrounds included in the studies, such as women who are Black, Asian, Latin American, Middle Eastern, and North African (MENA), as well as white marginalized groups, including Irish and Jewish women (Anitha, 2011; Burman & Chantler, 2005; Heron et al., 2021).

The definition of DVA service provider was broadly interpreted to cover a wide range of statutory and third-sector service needs and experiences, including housing support, immigration support, community-based services, helplines, therapeutic, psychological and rehabilitative support, and legal and financial matters.

Articles included within this review often referred to “culturally competent” or “culturally specific” services without specifying what this entails; to prevent the cultural framing of DVA, this review refers to these services as either intersectionally informed services or services specialized for minoritized women. It is important to note that, in the UK, any service aimed at supporting DVA “survivor–victims” is considered a specialist. Therefore, we refer to services that support DVA “survivor–victims” but are not specifically tailored to minoritized women as general specialist services. While general specialist DVA services may include support for minoritized women, the tailored and intersectionally informed approach provided by “by and for” services is unique. This review considers general specialist services and “by and for” services as distinct yet complementary facets of specialist service provision.

### Protocol and Registration

One author drafted a research protocol using a piloted template, which was discussed and revised with other co-authors. This final protocol was registered prospectively with the Open Science Framework on September 7, 2024 (https://osf.io/5vqh8 ).

### Eligibility Criteria

Articles for inclusion had to concern (a) Black and minoritized women (16 years or older), (b) any DVA service or support provided for Black and minoritized women, and (c) UK-based services. Articles reporting on the experiences of White women or Black and minoritized children or men were included if they provided comparative insights relevant to adult minoritized women’s experiences. Gray literature published by DVA specialist organizations was also included to identify relevant reports from smaller agencies dedicated to Black and minoritized service delivery in the UK.

Articles were excluded if they were (a) published before 1st January 2000 to maintain the review’s contemporary relevance, (b) non-peer-reviewed publications (except for relevant gray literature from DVA specialist organizations), (c) non-empirical publications (e.g. summaries of conference proceedings, commentaries, notes to the editor) when not published by an established DVA agency or organization, (d) focusing exclusively on support for perpetrators or (e) not written in English.

### Literature Search

The following databases were searched: MEDLINE, PsychInfo, and Academic Search Premier. The search, executed in June 2024, used the following terms:

DVA service search terms: “domestic violence services” OR “domestic violence support” OR “refuge” OR “by and for” AND “cultural competence” OR “cultural literacy” OR “socio-cultural factors” OR “specialist” OR “generalist” OR “mainstream”.

Population search terms: “ethnic minorities” OR “racial minorities” OR “ethnic groups” OR “minority groups” OR “BAME” OR “BME” OR “BMER” OR “MENA” OR “Black” OR “Asian” OR “North Africa” OR “Middle East” OR “ethnicity” OR “marginalised” OR “marginalized” OR “minoritised” OR “minoritized”.

Location search terms: “United Kingdom” OR “UK” OR “England” OR “Wales” OR “Scotland” OR “Northern Ireland”.

The terms “intimate partner violence”, “HBA”, “forced marriage” and “FGM” replaced the domestic violence terms in additional searches, as these often concern DVA. Search limiters included English language and publication dates (January 1, 2000 and June 31, 2024). Reference lists of returned publications were also used to identify additional sources.

The UK-based DVA organizations’ websites were searched for relevant gray literature including Women’s Aid and SafeLives. Alongside key general specialist providers, particular attention was paid to “by and for” services including Southall Black Sisters, Iranian and Kurdish Women’s Rights Organisation (IKWRO), Ashiana, Chinese Information and Advice Centre (CIAC), Latin American Women’s Rights Service (LAWRS), Latin American Women’s Aid, Imkaan, Jewish Women’s Aid, and Black Association of Women Step Out (BAWSO). When a search bar feature was available, reports and publications pages were searched using the keywords reported above; otherwise, website pages listing publications and reports were hand-searched for relevant reports.

Search results were uploaded to Mendeley and citations were exported into Rayyan, where duplicates were removed.

### Eligibility Assessment

All titles and abstracts were screened against the inclusion and exclusion criteria by one reviewer, and a second reviewer independently screened 20% of the sample, achieving a 99% agreement. The following full-text review was completed by one reviewer and checked by a second independent reviewer. Disagreements were resolved through discussion, involving a third review when necessary.

### Data Charting Process

A data extraction form was developed in Excel, reviewed, and iteratively refined by the research team. The form collected information on the authors, participant characteristics, ethnicity group of focus, and the key findings and conclusions pertaining to our research question.

### Thematic Analysis

Following Levac et al.’s (2010) enhancement of Arksey and O’Malley’s (2005) methodology, thematic analysis was conducted using Braun & Clarke’s (2006) recommended steps by familiarizing ourselves with the data, generating codes, searching for themes, reviewing themes, defining and naming the key themes, and producing the report. This review reports on the most prominent themes identified during the coding process. NVivo 13/ 2020 R1 (Lumivero, 2020) was used to conduct the thematic analysis. To ensure the trustworthiness of our data analysis, peer debriefing was used to discuss and review the key themes. Triangulation was achieved by incorporating multiple data sources, including grey literature and peer-reviewed studies while maintaining a detailed audit trail to enhance transparency and reproducibility. These measures collectively ensure the reliability and credibility of our findings.

## Results

### Selection of Sources of Evidence

**Figure 1.**
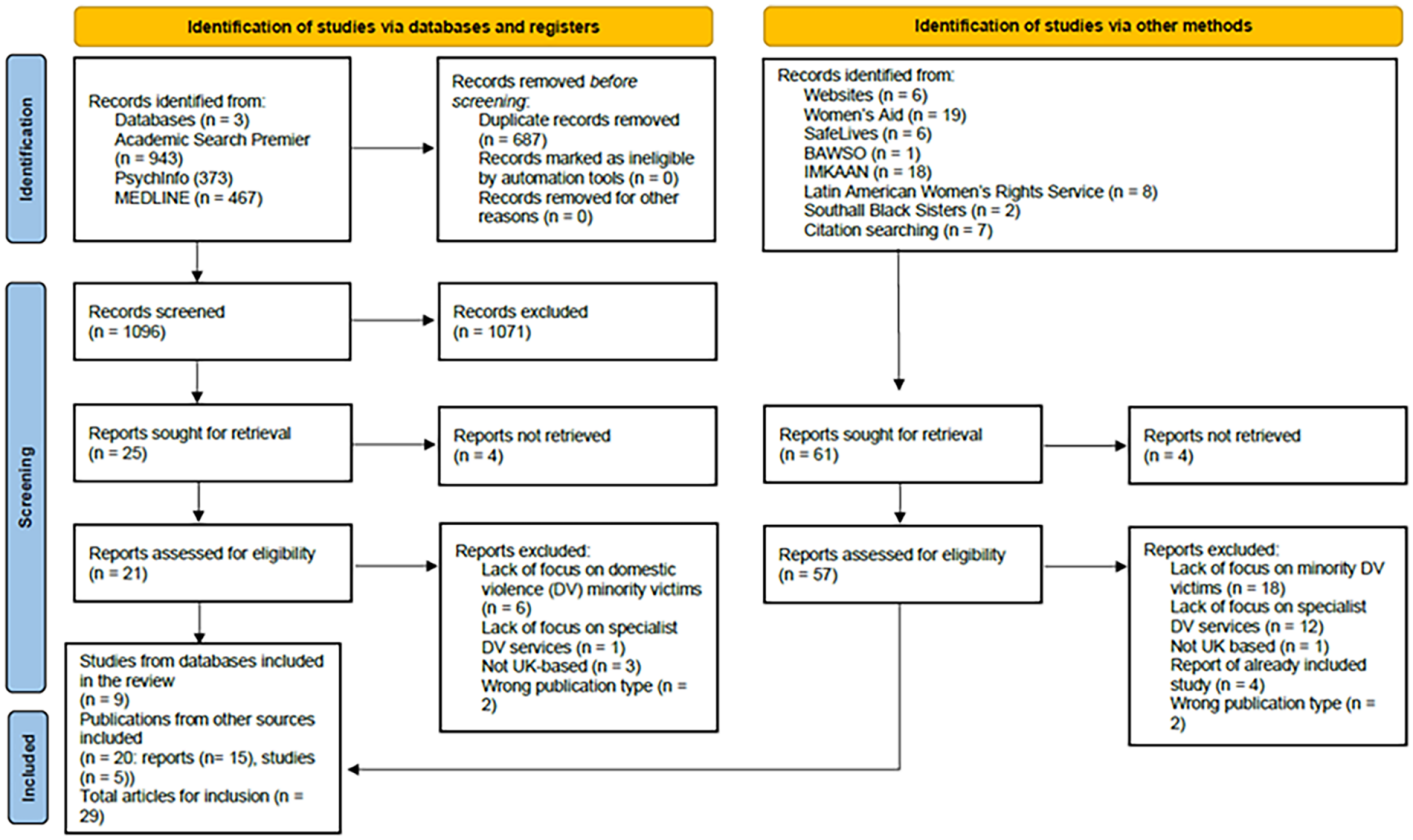
PRISMA flowchart for the study selection process in the scoping review. From Page et al. (2021).

The initial search identified 1,844 articles. After removing duplicates in Rayyan, 1,157 articles remained for screening. After title and abstract screening, 25 peer-reviewed articles remained, and, after full-text screening, 14 articles met the inclusion criteria. All 61 gray literature reports underwent full-text review, due to the absence of abstracts, and 15 reports were included. The final number of included articles was 29. The complete process is displayed in Figure 1.

### Characteristics of Sources of Evidence

Tables 1 and 2 present the characteristics of the included studies. Out of 29 studies included, 22 (76%) used qualitative methods, 3 (10%) used both qualitative and quantitative methods, and 4 (14%) used quantitative methods. Notably, all peer-reviewed studies were qualitative.

**Table 1.** Data Extraction of Peer-Reviewed Included Articles.

Reference (Year)	Methodology	Participants	Ethnicity referred to	Key Findings and Conclusions Pertaining to the Research Question
Heron et al. (2022)	Qualitative, semi-structured interviews	8 Black or Asian, 12 White	BAME	Faith-based resources as uniquely important for minoritized women Culturally sensitive services are needed
Heron et al. (2021)	Qualitative, semi-structured interviews, content analysis	21 White, 4 Black Caribbean, 2 Mixed, 1 African, 1 Pakistani	BAME	Language barriers and faith-based help-seeking behaviors are unique to minoritized women Culturally sensitive services are needed
Graca (2015)	Qualitative, semi-structured interviews in Portuguese	24 Portuguese women	Portuguese	Need for practical support Language barriers
Belur (2008)	Qualitative fieldwork observations in 2 police forces and 12 semi-structured interviews	BME police officers and representatives from BME communities	BME	Language and community-related barriers Prejudice and discrimination within services
Humphreys (2007)	Qualitative, Narrative review	N/A	BAME	Poverty and ethnicity intersect to create many specific barriers to help-seeking Language barriers Prejudice and discrimination within services
Magill (2022)	Qualitative, in-depth semi-structured interviews	4 employees from Southall Black Sisters	BAME	Barriers for NRPF women Importance of community aspects of services for minoritized women Language barriers Poverty and ethnicity intersect to create many specific barriers to help-seeking
Anitha (2011)	Qualitative, semi-structured interviews, thematic analysis	30 women from India, Pakistan, and Bangladesh	South Asian	Funding barriers for NRPF women Prejudice and discrimination within services
Burman and Chantler (2005)	Qualitative, semi-structured interviews	23 African, Caribbean, Irish, Jewish, and South Asian victims	Minoritized women	Cultural “respect” to excuse non-intervention Funding barriers for NRPF women
Sultana et al. (2022)	Qualitative, Systematic review	N/A	South Asian	Community-related barriers Barriers for NRPF women
Istratii et al. (2024)	Qualitative, Scoping review	N/A	BAME	Poor religious understanding within mainstream services
Burman et al. (2004)	Qualitative, interviews	26 service providers and 23 DVA victims	BAME	Cultural “respect” to excuse non-interventions Confidentiality concerns for staff members of same ethnicity Culturally competent practices must be adapted within mainstream services
Gill and Banga (2008)	Qualitative, open-ended unstructured questionnaire	37 service providers in the social and domestic violence sector	BAME	Intersectional framework important within services Community aspects of “by and for” services as important Cost-effectiveness of “by and for” services
Femi-Ajao et al. (2018)	Qualitative, systematic review, thematic analysis	N/A	BAME	Language and immigration barriers Prejudice and discrimination within services Community and religious sources as important to minoritized women
Styles (2010)	Qualitative, semi-structured interviews	12 women working in specialized DV services	BME	Culturally competence within specialized services Lack of cultural understanding within mainstream services

**Table 2.** Data Extraction of Included Gray Literature Articles.

Reference (Year)	Methodology	Participants	Ethnicity referred to	Key Findings and Conclusions
Lidubwi et al. (2024)	Mixed methods, scoping review, case study, focus groups, and surveys	Former BAWSO users and current staff	BME	Prejudice and discrimination within services Language barriers Funding issues for NRPF women
Roy et al. (2008)	Qualitative, interviews, questionnaires, and case study	12 local authorities, 15 refuges, and two legal specialists	BAMER	Language barriers Prejudice and discrimination within services Funding issues for NRPF women and “by and for”
Mouj and Imkaan (2008)	Qualitative, discussion with service providers	N/A	BAMER	Lack of recognition of “by and for” Funding issues for “by and for”
Imkaan and Rights of Women (2016)	Qualitative, questionnaires, discussion workshops	20 refuges, 15 criminal justice agents, and 14 Local Authorities	BAMER	Prejudice and discrimination within services Funding barriers Assumptions of culture
Imkaan (2020)	Qualitative, two surveys, interviews	Imkaan’s network	BAME	Funding barriers for “by and for” especially concerning COVID-19
Thiara and Roy (2010)	Quantitative, secondary data analysis, pilot project	10 service providers and 124 service users	BAMER	Importance of “by and for” for minoritized women Community aspect of “by and for”
Thiara and Roy (2012)	Quantitative, secondary data analysis, pilot project	10 service providers, 183 women	BAMER	“By and for” important for minorities Community aspect of “by and for” Language support from “by and for”
ASCENT and Imkaan (2016)	Qualitative, report of an event	N/A	BME	Intersectional framework of “by and for” services Funding barriers for “by and for”
Thiara et al. (2020)	Qualitative, interviews, case study	36 women	BME	Assumption of culture Community aspect of “by and for” Intersectional framework of “by and for”
McIlwaine et al. (2019)	Mixed methods, survey, interviews, focus groups	60 migrant women and 10 service staff	BME	Language barriers Prejudice and discrimination within services Funding barriers for “by and for”
Sheil et al. (2024)	Quantitative, cost-benefit analysis	N/A	BAME	Cost-effectiveness of “by and for” Funding barriers for “by and for” Language support and holistic services
Women’s Aid (2023)	Quantitative, pathways and cost-benefit analysis	N/A	BAMER	Cost-effectiveness of “by and for” Funding barriers for “by and for” “By and for” important for minorities
McCarthy et al. (2022)	Mixed methods, survey, focus groups	228 survey respondents, 2 focus groups	BME	Holistic mental health support within “by and for” “By and for” important for minorities
Women’s Aid (2022)	Qualitative, inquiry hearings	N/A	BME	Language support within “by and for” Holistic mental health support within “by and for”
Thiara and Harrison (2021)	Qualitative, literature review	N/A	BME	Mental health support within “by and for” as holistic Intersectional framework within “by and for” Community aspect of “by and for”

*Note.* NRPF = No recourse to public funds.

The methodologies of the peer-reviewed studies were as follows: 9 (64%) used interviews, 4 (29%) were literature reviews, and 1 (7%) used a questionnaire. Of the non-peer-reviewed publications, the methods were diverse: 6 (40%) used interviews and focus groups, 2 (13%) reported a cost-benefit analysis, 3 (20%) were reports of and questionnaires from events with practitioners, 2 (13%) were based on secondary data analysis, 1 (7%) was a narrative literature review, and 1 (7%) was a report of discussions with “by and for” service providers.

Regarding the focus on minoritized communities, 26 (90%) articles referred to overarching minoritized groups such as Black, Asian, and Minoritized Ethnic groups (BAME) or Black and Minoritized Ethnic Groups (BME). For consistency, this review refers to these groups as Black and minoritized. Additionally, 1 (3%) article focused on Portuguese women, and 2 (7%) on South Asian women.

### Key Findings

**Figure 2.**
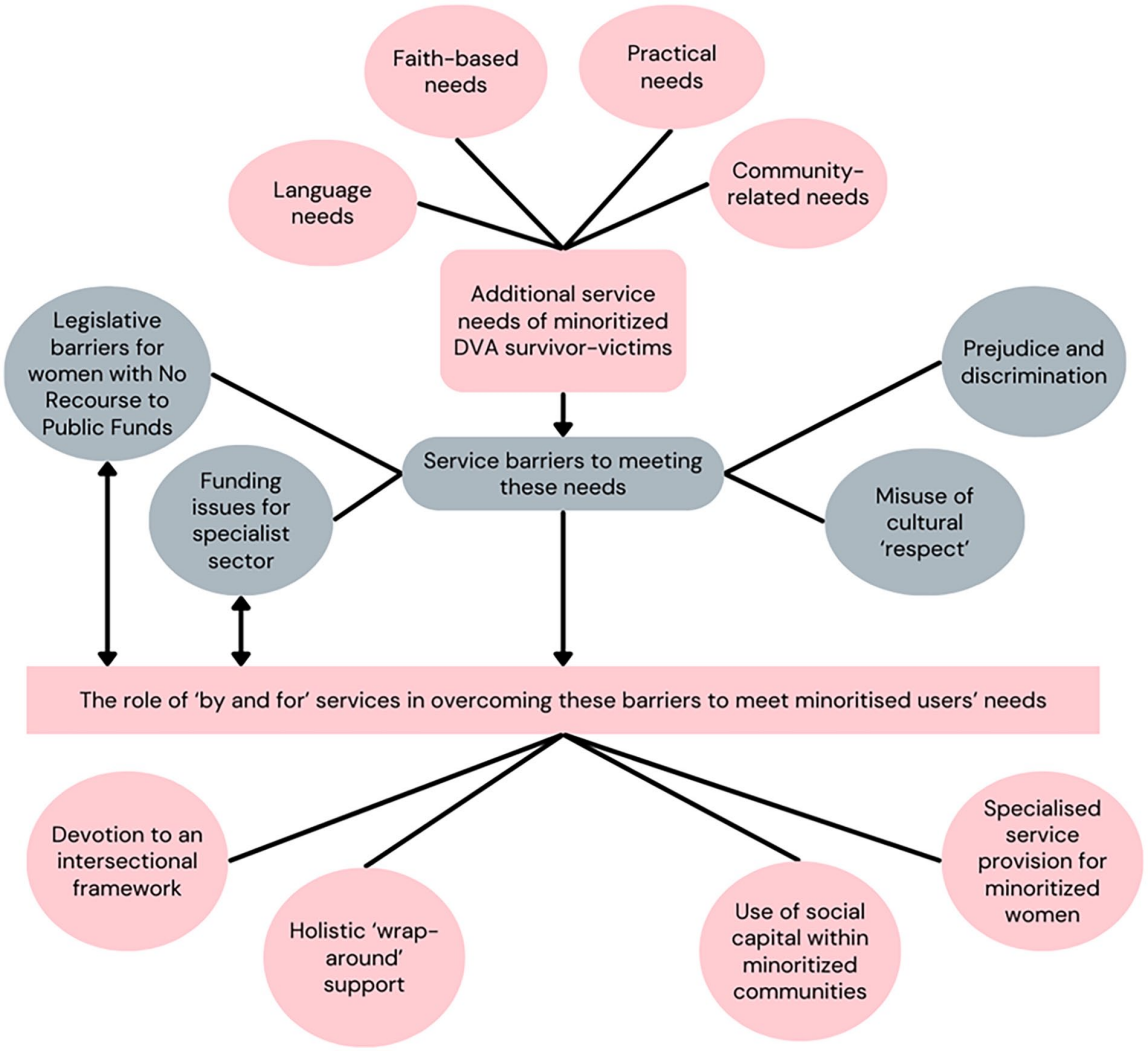
Flowchart of minoritized women’s experiences of approaching DVA services with additional needs, barriers to meeting needs, and the role of “by and for” services in overcoming barriers.

Analysis of the included articles revealed three critical themes related to the experiences of service provision for Black and minoritized DVA “survivor–victims” in the UK: additional service needs, barriers to meeting needs, and the role of “by and for” services in overcoming barriers. The following thematic map is displayed in Figure 2.

### Theme 1: Additional Service Needs

All 29 included articles noted the presence of additional support needs for Black and minoritized “survivor–victims” of DVA, with the need for language support, faith-based resources, community-related needs, and practical support most commonly cited (All-Party Parliamentary Group [APPG] & Women’s Aid, 2022; Anitha, 2011; ASCENT & Imkaan, 2016; Belur, 2008; Burman et al., 2004; Burman & Chantler, 2005; Femi-Ajao et al., 2018; Gill & Banga, 2008; Graca, 2015; Heron et al., 2022; Heron et al., 2021; Humphreys, 2007; Imkaan, 2020; Imkaan & Rights of Women, 2016; Istratii et al., 2024; Lidubwi, 2024; Magill, 2022; McCarthy et al., 2022; McIlwaine et al., 2019; Mouj & Imkaan, 2008; Roy & Imkaan, 2008; Sheil et al., 2024; Styles, 2010; Sultana et al., 2022; Thiara & Harrison, 2021; Thiara & Roy, 2009, 2012, 2020; Women’s Aid, 2023). This review reports the most commonly mentioned support needs throughout the included articles; however, it is pivotal to maintain that DVA experiences vary individually, and Black and minoritized women’s experiences must not be oversimplified or generalized.

### Need for Language Support

Language needs were identified as often evident for Black and minoritized “survivor–victims” in 16 out of 29 articles (Belur, 2008; Femi-Ajao et al., 2018; Graca, 2015; Heron et al., 2022; Humphreys, 2007; Lidubwi, 2024; Magill, 2022; McCarthy et al., 2022; Mouj & Imkaan, 2008; Roy & Imkaan, 2008; Styles, 2010; Sultana et al., 2022; Thiara & Harrison, 2021; Thiara & Roy, 2009, 2012; Thiara & Roy, 2020). Non-fluent English speakers struggled to have their needs and experiences heard and understood, often facing impatience from agency staff (Lidubwi, 2024). Limited access to dialect-appropriate interpreting and inappropriate choices of interpreters exacerbated these difficulties (Belur, 2008; Femi-Ajao et al., 2018; Magill, 2022; Mouj & Imkaan, 2008). Language barriers can also lead to isolation from the wider community (Graca, 2015), increasing the likelihood of entrapment in abusive relationships (Humphreys, 2007).

### Need for Faith-Based Resources

The need for faith-based resources was identified as unique to Black and minoritized communities in 6 out of 29 articles (Femi-Ajao et al., 2018; Heron et al., 2021, 2022; Istratii et al., 2024; Lidubwi, 2024; Thiara & Roy, 2012). Religious practices were reported as a source of strength-building and a precursor to seeking formal support (Femi-Ajao et al., 2018; Heron et al., 2021; Istratii et al., 2024). However, Thiara and Roy (2012) noted that support from religious organizations was often unhelpful due to pressure to remain in the relationship. Challenges to collaboration between DVA services and religious organizations must be addressed to support religious needs of Black and minoritized “survivor–victims” (Istratii et al., 2024; Lidubwi, 2024).

### Need for Community-Related Support

Unique needs and fears of minoritized “survivor–victims” concerning their social community were reported in 5 out of 29 articles (Burman et al., 2004; Femi-Ajao et al., 2018; Sultana et al., 2022; Thiara & Roy, 2009, 2012, 2020). Minoritized “survivor–victims” expressed concerns about bringing dishonor to or facing retaliation from their community when reporting DVA (Femi-Ajao et al., 2018; Sultana et al., 2022; Thiara & Roy, 2020). Burman et al. (2004) found that the strong emphasis on family and community honor was unique to minoritized communities. While these articles highlighted the negative implications of communities on DVA experience, Thiara & Roy (2009, 2012) found potential social capital for help-seeking within minoritized communities, with “survivor–victims” likely to talk to family and friends before seeking formal support.

### Need for Practical Support

Minoritized peoples’ need and preference for practical support, such as support regarding housing, finance, and child protection, was identified in 4 out of 29 articles (Graca, 2015; Humphreys, 2007; McCarthy et al., 2022; Styles, 2010). While practical support is needed for all “survivor–victims”, minoritized “survivor–victims” often have an intensified need for practical support (Graca, 2015; McCarthy et al., 2022). Financial independence and employment are particularly challenging to achieve when experiencing language and immigration barriers (Humphreys, 2007; Styles, 2010). Before achieving employment and stable housing, “survivor–victims” can find services such as counseling unhelpful (McCarthy et al., 2022). Minoritized “survivor–victims” have reported avoiding voluntary sector services, perceiving them as lacking in practical support (Graca, 2015).

### Theme 2: Additional Service Barriers to Accessing Effective Support

Once Black and minoritized “survivor–victims” access DVA services, additional barriers to their recovery become evident; the most prominent sub-themes were prejudice or discrimination within general specialist services, legislative restrictions on support for women with No Recourse to Public Funds (NRPF), the misuse of cultural “respect” as an excuse for non-intervention, and funding issues.

#### Prejudice and Discrimination

A significant barrier was the prejudiced and discriminatory attitudes found within general specialist DVA services, reported by 16 out of 29 articles (APPG & Women’s Aid, 2022; Anitha, 2011; Belur, 2008; Burman et al., 2004; Burman & Chantler, 2005; Femi-Ajao et al., 2018; Imkaan & Rights of Women, 2016; Istratii et al., 2024; Lidubwi, 2024; McCarthy et al., 2022; Mouj & Imkaan, 2008; Roy & Imkaan, 2008; Sultana et al., 2022; Thiara & Harrison, 2021; Thiara & Roy, 2009, 2020). The prejudices and stereotypes were described as stemming from a lack of engagement with minoritized communities, exacerbated by funding restraints that prevent adequate staff training or interpreting services resulting in discriminatory responses (Belur, 2008; Sultana et al., 2022,). Poor understanding of the intersectional needs of minoritized DVA “survivor–victims” results in inappropriate and ineffective support (APPG & Women’s Aid, 2022). For example, a lack of understanding of the impact of family and community structures within South Asian communities led to disbelief and insensitivity in responses to DVA committed by the wider family (Anitha, 2011; Belur, 2008; Lidubwi, 2024; Sultana et al., 2022). Additionally, the insensitivity to understanding family and community structures within certain groups further isolates “survivor–victims” from seeking help (Burman et al., 2004; Burman & Chantler, 2005; Femi-Ajao et al., 2018; McCarthy et al., 2022; Thiara & Harrison, 2021; Thiara & Roy, 2020).

#### Legislative Barriers for Women with Insecure Immigration Status and No Recourse to Public Funds

The legislative landscape in the UK presents significant challenges for migrant women with insecure status and NRPF, as noted in 14 out of 29 articles (Anitha, 2011; Belur, 2008; Burman & Chantler, 2005; Femi-Ajao et al., 2018; Lidubwi, 2024; Magill, 2022; McCarthy et al., 2022; McIlwaine et al., 2019; Mouj & Imkaan, 2008; Roy & Imkaan, 2008; Sheil et al., 2024; Styles, 2010; Sultana et al., 2022; Thiara & Harrison, 2021). The NRPF stipulation restricts individuals subject to immigration control from accessing public funds, such as welfare benefits or housing assistance. Those unable to prove that their relationship ended due to DVA are not recognized and supported by the UK government (Lidubwi, 2024; Roy & Imkaan, 2008; Thiara & Harrison, 2021). As a result, NRPF women are frequently turned away from refuges, forcing them to return to their country or to abusive relationships where they are at risk of further violence (Anitha, 2011; Belur, 2008; Mouj & Imkaan, 2008). Additionally, negative perceptions towards immigrant populations in the UK further impact support decisions for NRPF women (Roy & Imkaan, 2008). General specialist DVA services often lack critical engagement with immigration issues, resulting in “by and for” services disproportionately bearing the responsibility for NRPF service provision, straining an already under-funded sector (Burman & Chantler, 2005; Sheil et al., 2024; Thiara & Roy, 2009).

#### Cultural “Respect” as an Excuse for Non-Intervention

The misuse of cultural “respect” as an excuse for non-intervention was another barrier, reported in 8 out of 29 articles (Anitha, 2011; Belur, 2008; Burman et al., 2004; Burman & Chantler, 2005; Thiara & Roy, 2020; Humphreys, 2007; Istratii et al., 2024; Lidubwi, 2024). DVA is sometimes perceived as a cultural practice, leading general specialist service providers to attribute non-intervention to cultural “respect” (Anitha, 2011; Belur, 2008; Burman & Chantler, 2005; Lidubwi, 2024). Burman et al. (2004) argue that accepted attitudes of privacy towards DVA within minoritized communities excuse non-intervention, leave “survivor–victims” further silenced, and remove accountability from service providers. These patterns of cultural “respect” are rooted in the cultural framing of DVA rather than considering the complex intersecting factors that lead to the victimization of minoritized women (Anitha, 2011; Istratii et al., 2024; Lidubwi, 2024; Thiara & Roy, 2020).

#### DVA Sector Funding Issues

Funding remains a consistent barrier to adequate service provision throughout the specialist DVA service sector, as highlighted by 8 out of 29 articles (APPG & Women’s Aid, 2022; Burman & Chantler, 2005; Gill & Banga, 2008; Imkaan & Rights of Women, 2016; Lidubwi, 2024; Magill, 2022; Mouj & Imkaan, 2008; Roy & Imkaan, 2008). Historically, service provision for women and children experiencing DVA has been underfunded, with specialist services for minoritized women being particularly neglected (APPG & Women’s Aid, 2022; Burman & Chantler, 2005; Magill, 2022; Mouj & Imkaan, 2008; Roy & Imkaan, 2008). Mouj and Imkaan (2008) highlight that “by and for” services are often overlooked in funding decisions due to assumptions that general specialist services can adequately address the complex needs of minoritized service users. Consequently, minoritized women are disproportionately turned away from refuges, mental health support, and social housing (APPG & Women’s Aid, 2022; Magill, 2022). Recommendations for improving service provision call for long-term, holistic protected funding allocated to specialist DVA organizations for minoritized “survivor–victims” (APPG & Women’s Aid, 2022; Gill & Banga, 2008; Lidubwi, 2024). The cost-effectiveness of “by and for” services is supported by evidence from 4 out of 29 articles; non-specialist services leave the needs of minoritized women unmet, leading to further financial strain on alternative public services such as the National Health Service (ASCENT & Imkaan, 2016; Gill & Banga, 2008; Sheil et al., 2024; Women’s Aid, 2023).

### Theme 3: The role of “by and for” services in overcoming these barriers

The literature has noted multiple strengths of the “by and for” approach. These include increasing access to services specialized for minoritized women, an intersectional framework, holistic “wrap-around” services, and utilization of the social capital within minoritized communities. The need for incorporating support in general specialist services for Black and minoritized women who are hesitant to access “by and for” services was noted.

#### Service Provision Specialized for Black and Minoritized Women

Specialized service provision is crucial for minoritized “survivor–victims”, as noted by 14 out of 29 articles (APPG & Women’s Aid, 2022; Burman & Chantler, 2005; Gill & Banga, 2008; Heron et al., 2022; Istratii et al., 2024; Lidubwi, 2024; McCarthy et al., 2022; McIlwaine et al., 2019; Styles, 2010; Sultana et al., 2022; Thiara & Roy, 2009, 2012, 2020; Women’s Aid, 2023). These services are tailored to overcome the barriers of prejudice and discrimination and the misuse of cultural “respect” within general specialist service provision. Practitioners from minoritized backgrounds are more likely to be understanding and empathetic towards minoritized experiences, fostering a supportive environment (Gill & Banga, 2008; Lidubwi, 2024; Sultana et al., 2022). In various studies, service users reported preferring “by and for” support due to several reasons: connection with other “survivor–victims” from similar backgrounds, sense of safety, ability to communicate in their own language, feeling heard, and intersectionally informed responses from staff (Burman & Chantler, 2005; Gill & Banga, 2008; Lidubwi, 2024; McCarthy et al., 2022; Thiara & Roy, 2009, 2012, 2020).

#### Intersectional Framework

Understanding intersectional experiences and using an intersectional framework to address DVA in minoritized communities is another strength noted by 12 out of 29 articles (Anitha, 2011; ASCENT & Imkaan, 2016; Burman et al., 2004; Burman & Chantler, 2005; Femi-Ajao et al., 2018; Gill & Banga, 2008; Humphreys, 2007; Istratii et al., 2024; Lidubwi, 2024; Magill, 2022; McIlwaine et al., 2019; Thiara & Roy, 2020). Intersectionality has been described as the most accurate framework for understanding the complex barriers and needs that minoritized “survivor–victims” face (ASCENT & Imkaan, 2016; Burman & Chantler, 2005; McIlwaine et al., 2019). Without a grounding in intersectionality, services risk essentializing and pathologizing culture (Anitha, 2011; Burman et al., 2004; Humphreys, 2007; Istratii et al., 2024). ASCENT & Imkaan (2016) describe how “by and for” services embed their intersectional framework throughout the entire organization and individual practice; a reflective and proactive approach addresses power imbalances and inequalities within the agency, ensuring minoritized experiences are central to service provision. Through a centering of an intersectional framework, “by and for” services are uniquely placed to meet the intersectional needs of minoritized DVA service users (Burman & Chantler, 2005; Gill & Banga, 2008; Lidubwi, 2024).

#### Holistic “Wrap-Around” Support

The strength of the holistic “wrap-around” support provided by “by and for” organizations was noted by 10 out of 29 articles (APPG & Women’s Aid, 2022; Belur, 2008; Graca, 2015; Imkaan, 2020; Imkaan & Rights of Women, 2016; McCarthy et al., 2022; Sheil et al., 2024; Thiara & Harrison, 2021; Thiara & Roy, 2012, 2020). “By and for” services provide comprehensive and wide-ranging holistic support by applying non-Eurocentric interventions and support methods specifically developed for their service users (Thiara & Roy, 2020). Some types of support have been reported as particularly effective when administered in a “by and for” service setting. For example, mental health support provided in “by and for” settings has been praised for its trauma-informed provision alongside the availability of alternative and talking therapies (APPG & Women’s Aid, 2022; McCarthy et al., 2022; Thiara & Harrison, 2021; Thiara & Roy, 2020). This flexible, trauma-informed response is best suited to meet the needs of minoritized women experiencing intergenerational trauma from repeated victimization rooted in race and gender (Thiara & Harrison, 2021). Furthermore, “by and for” services better facilitate service users’ language needs by enabling non-English communication with staff and counselors without needing an interpreter (APPG & Women’s Aid, 2022; Imkaan, 2020; Imkaan & Rights of Women, 2016; McCarthy et al., 2022; Sheil et al., 2024; Thiara & Roy, 2020). Belur (2008) described how DVA “survivor–victims” were more comfortable speaking in their first language, even if they knew English, due to the emotional complexity of describing their DVA experience. The ability to communicate in their own language directly with staff was reported as a critical factor in service satisfaction for Black and minoritized women (Graca, 2015; Thiara & Roy, 2012).

#### Utilizing Social Capital within Minoritized Communities

Despite evidence suggesting the negative impact of community values of honor on DVA experience (Burman et al., 2004; Femi-Ajao et al., 2018; Sultana et al., 2022; Thiara & Roy, 2020), 3 out of 29 articles noted how “by and for” services recognize the benefits of utilizing existing social capital within minoritized communities (Imkaan, 2020; McCarthy et al., 2022; Thiara & Harrison, 2021; Thiara & Roy, 2012). Space to reconnect with other “survivor–victims” from the same background is highly valued in forming new coping strategies and support systems (Thiara and Roy, 2020; Thiara & Harrison, 2021). McCarthy et al. (2022) reported that minoritized “survivor–victims” requested more support groups and opportunities to meet “survivor–victims” from similar backgrounds to aid their mental and social recovery. “By and for” services provide this space for reconnection by being deeply connected with the local community (Imkaan, 2020). Thiara and Roy (2012) found that service users preferred “by and for” support, partly attributed to connecting with other “survivor–victims” from similar backgrounds. “By and for” services’ emphasis on community-level engagement is pivotal in providing social support for Black and minoritized “survivor–victims”.

#### Hesitancy Towards “By and For” Services

However, 2 out of 29 articles reported hesitancy towards “by and for” services among some Black and minoritized “survivor–victims”. Burman et al. (2004) and Thiara and Harrison (2021) reported that confidentiality fears with practitioners from the same ethnic background may result in a reluctance to approach “by and for” services. General specialist DVA services need to develop interventions specialized for these Black and minoritized “survivor–victims” who are more comfortable approaching these services. However, Istratii et al. (2024) emphasized that recognizing the need for general specialist DVA services to incorporate intersectionally-informed support must not be misconstrued as an equal replacement for “by and for” service provision; the intersectional leadership structure of “by and for” services is unique, best situating themselves to meet the intersectional needs of specific minoritized groups (Imkaan, 2018).

## Discussion

This scoping review aimed to map the current understanding of Black and minoritized women’s experiences of specialist DVA services in the UK. Our findings reveal significant insights from both academic and gray literature, situating UK-based studies within the broader context of the increasing recognition of specialist DVA services for minoritized women. These findings are summarized in Table 3. The review underscores the variation in experiences among Black and minoritized DVA “survivor–victims”, emphasizing that these differences should not be viewed as inherently cultural. Instead, culture intersects with other structural inequalities, such as immigration status, institutional racism, colonialism, class, disability and sexuality, to further disadvantage Black and minoritized “survivor–victims” (Anitha, 2011; ASCENT & Imkaan, 2016; Burman et al., 2004; Burman & Chantler, 2005; Femi-Ajao et al., 2018; Gill & Banga, 2008; Humphreys, 2007; Istratii et al., 2024; Lidubwi, 2024; Magill, 2022; McIlwaine et al., 2019; Thiara & Roy, 2020). Focusing solely on cultural explanations for issues impacting minoritized communities can lead to harmful stereotypes and overlook the complex, intersecting inequalities that contribute to these problems (JAS, 2024).

**Table 3.** Summary Table of the Key Findings Within This Review.

Critical Findings
Black and minoritized “survivor–victims” of DVA approach services with additional support needs, such as language, faith-based, practical, and community-related needs.
Multiple barriers exist to meeting these needs within specialist generalist and services specialized for minoritized women.
Specialist generalist services can feature prejudice, discrimination, and misuse of cultural respect to excuse non-intervention.
Specialist service provision for Black and minoritized DVA “survivor–victims” is particularly impacted by restrictive legislation on the support and funds available to women with No Recourse to Public Funds, as well as experiencing disproportionate underfunding.
Specialist services in the UK, specifically “by and for” services—those run by and for Black and minoritized people—effectively supported and provided services for Black and minoritized “survivor–victims”. This was due to their devotion to an intersectional framework, holistic “wrap-around” service provision, usage of the social capital within minoritized communities, and provision of support specific to minoritized groups.

### The Role of Specialist Services for Black and Minoritized Women

Our results indicate that “by and for” specialist services are better placed to address the needs of Black and minoritized “survivor–victims”, including language, practical, faith-based, and community-related needs. Minoritized “survivor–victims” can experience prejudice and discriminatory responses when DVA response frameworks are not informed by an intersectional approach, as well as facing restricted access to support due to funding issues and barriers for women with NRPF (Thiara, 2022). It is pivotal that stereotypes towards certain communities, such as “angry and strong” Black women, that result in discriminatory and potentially fatal service responses are challenged (Imkaan & Centre for Women’s Justice, 2023). DVA services tailored for Black and minoritized women are able to overcome these barriers to provide effective support (Imkaan, 2013), as evidenced in this review.

As Asante (2024) notes, the needs of Black and minoritized DVA “survivor–victims” are best met through an intersectional framework embedded into “by and for” practice (ASCENT & Imkaan, 2016). “By and for” services adopt a flexible, holistic approach, tailoring support to meet the distinct intersectional needs of Black and minoritized women (APPG & Women’s Aid, 2022; Belur, 2008; Graca, 2015; Imkaan, 2020; Imkaan & Rights of Women, 2016; McCarthy et al., 2022; Sheil et al., 2024; Thiara & Harrison, 2021; Thiara & Roy, 2012, 2020). For example, “by and for” services responded to language needs by enabling communication in service users’ native languages, enhancing service satisfaction (APPG & Women’s Aid, 2022; Imkaan, 2020; Imkaan & Rights of Women, 2016; McCarthy et al., 2022; Sheil et al., 2024; Thiara & Roy, 2020). Furthermore, “by and for” services recognize the value of community support, leveraging social capital for help-seeking and coping mechanisms rather than focusing solely on restrictive community norms (Imkaan, 2020; McCarthy et al., 2022; Thiara & Harrison, 2021; Thiara & Roy, 2012). This flexible tailored response is pivotal for effectively addressing the complex needs of Black and minoritized DVA “survivor–victims”, as evidenced by similar findings in US interventions (Fuchsel, 2014; Fuchsel & Hysjulien, 2013; Perilla et al., 2012). “By and for” services have increased the needs met for Black and minoritized DVA “survivor–victims” in the UK (ASCENT & Imkaan, 2016; Gill & Banga, 2008; Sheil et al., 2024; Women’s Aid, 2023).

### Gaps in the Understanding of Black and Minoritized Women’s Experiences of DVA Services

In addition to exploring Black and minoritized women’s experiences of specialist services, this review aimed to identify gaps in the current understanding of this topic. Regarding study characteristics, the overwhelming body of literature (76%) was qualitative, including all peer-reviewed studies. Despite the strength of qualitative methods within DVA research, it is crucial to employ a range of methodologies to comprehensively explore the needs and experiences of Black and minoritized DVA “survivor–victims”. Additionally, there was a notable lack of focus on individual minoritized groups, with 26 (96%) articles referring to overarching groups such as “BAME” or “BME.” This generalization often stems from sample size and funding constraints, masking the intersectional complexities different groups face. As Asante (2024) argues, the intersection of structural inequalities and cultural factors may manifest differently across Black and minoritized groups, highlighting the need for research that specifically explores the needs of individual communities. A significant gap evidenced by this review is the lack of nuanced engagement from peer-reviewed research with the role of specialist services in supporting Black and minoritized “survivor–victims”. The gray literature provided rich and nuanced insight into the lived experiences and needs of Black and minoritized “survivor–victims”.

Contrary to the gray literature, academic peer-reviewed literature has predominantly focused on identifying service needs and barriers to help-seeking without delving deeply into “survivor–victims”’ experiences of service providers. The peer-reviewed literature has surmised the need for intersectionally competent services from establishing unique service needs and barriers. Following this, peer-reviewed literature should develop an understanding of how existing specialist DVA services are experienced by Black and minoritized “survivor–victims”’ recovery. Academic literature has yet to engage with the role of “by and for” services within the UK DVA sector, with only two studies in this review referring specifically to “by and for” services (Istratii et al., 2024; Magill, 2022). There is a notable gap in recent studies, with only 13 out of 29 studies being published in the previous 5 years. Given the promising findings presented within this review, it is crucial that the key role of “by and for” services is recognized within the peer-reviewed literature.

### Strengths and Limitations

This scoping review adhered to the Arksey and O’Malley (2005) framework, enhanced by Levac et al.’s (2010) recommendations for integrating thematic analysis. Methodological rigor was maintained to map current literature and guide future research. However, some relevant literature may have been missed. To mitigate this, an advisory board of experts and researchers from the UK DVA specialist sector provided guidance to minimize the risk of missing relevant studies and gray literature reports, reducing selection bias. Regarding diversity, the articles included in this review lacked consideration of intersectionality beyond ethnicity, socioeconomic status, language, nationality, religion, and culture. Some included articles acknowledged the absence of data on the intersection of ethnicity with characteristics such as disability, gender identity, sexuality, or age (Roy & Imkaan, 2008; Thiara & Harrison, 2021; Thiara & Roy, 2012, 2020). Additionally, articles focusing solely on perpetrators, male “survivor–victims”, and children were beyond the scope of this review.

### Implications for Further Research

These findings highlight several areas for future research, as summarized in Table 4. These findings highlight several areas for future research. Firstly, the positive impact of “by and for” services on Black and minoritized DVA “survivor–victims” is evident within gray literature sources, and further academic research should explore the type of specialist support provided by “by and for” services. Specialist DVA organizations face disproportionate underfunding, impacting their service delivery; future research should assess the cost-effectiveness of these services to inform funding decisions. Additionally, integrating both qualitative and quantitative methodologies will provide a comprehensive understanding of the lived experiences of “by and for” services for Black and minoritized women “survivor–victims”.

The “by and for” sector exemplifies effective service delivery for Black and minoritized women. The embedded intersectional framework implemented may provide an innovative service framework that can be developed within other contexts for supporting Black and minoritized “survivor–victims”. Future research should expand beyond the facilitators and barriers of accessing DVA services to examine the effectiveness of ongoing support. While developing our understanding of help-seeking behaviors is vital, this review highlights a gap in research on the effectiveness of support provided beyond initial contact.

**Table 4.** Implications of This Review’s Findings on Policy, Practice, and Further Research.

Implications for Further Policy, Practice, and Research
The positive impact of specialist services, specifically “by and for,” was highlighted throughout gray literature articles, with less nuanced engagement from peer-reviewed literature. Replication of the promising findings within non-peer-reviewed literature is needed to fully consider the lived experiences and needs of Black and minoritized women within academic discourse.
To address the limited empirical research on certain subgroups, such as the disabled, those with insecure migrant status, and older minoritized women, future research agendas should prioritize studies that examine these intersections within the DVA context.
Policymakers, funding bodies, and researchers should encourage diverse methodological approaches, including quantitative studies, and peer-reviewed evidence, to generate a comprehensive understanding of how “by and for” services operate and contribute to service improvement for Black and minoritized women.
Policymakers should implement standardized monitoring and evaluation frameworks that systematically collect demographic data on ethnicity, disability, sexual orientation, and age within DVA service use. Such data collection is vital for identifying service gaps and ensuring accountability. Evaluation efforts should assess service efficacy, accessibility, and outcomes for Black and minorized women, ensuring policy adjustments are data-informed and responsive to evolving needs.
Policies should prioritize funding and resource allocation for “by and for” services specifically tailored to Black and minoritized women, which address intersectionally informed needs and promote accessibility. These services, led by individuals with shared backgrounds and experiences, have proven critical in overcoming structural barriers and providing effective support, particularly in language assistance and community engagement.
Service providers and practitioners, when working with minoritized “survivor–victims” of DVA, must be trained in and follow intersectional approaches to provided nuanced and contextualized understanding, without relying on cultural assumptions surrounding DVA.
The importance of services specialized for minorized women must be recognized within specialist and generalist services, fostering collaboration and understanding between service providers. Understanding the critical role of “by and for” services and recognizing the specific barriers faced by minoritized groups are key to improving the response of generalist providers and ensuring a cohesive, respectful, and effective support system.
Policymakers and service providers must urgently adopt intersectionally informed frameworks in DVA responses. Recognizing the compounded challenges that arise from intersecting identities—such as race, disability, sexuality, and age—can foster a more inclusive service approach. This would involve systematically integrating intersectional perspectives into service design and implementation, ensuring more equitable access and tailored support for minoritized women.

## Conclusion

This scoping review highlights the critical need for specialist DVA services for Black and minoritized women in the UK. Our findings underscore the effectiveness of the “by and for” service sector in meeting these women’s unique needs through intersectional approaches. However, significant gaps remain, and academic peer-reviewed literature needs to replicate and explore the experiences detailed within gray literature reports. The “by and for” sector should be recognized within peer-reviewed literature to explore effective service delivery for Black and minoritized DVA “survivor–victims”.
